# New onset Graves' disease as a cause of an adrenal crisis in an individual with panhypopituitarism: brief report

**DOI:** 10.1186/1756-6614-1-7

**Published:** 2008-11-19

**Authors:** Krzysztof C Lewandowski, Magdalena Marcinkowska, Elżbieta Skowrońska-Jóźwiak, Jacek Makarewicz, Andrzej Lewiński

**Affiliations:** 1 Department of Endocrinology and Metabolic Diseases, Medical University, Lodz, Poland; 2 Department of Endocrinology, Polish Mother's Memorial Hospital – Research Institute, Lodz, Poland

## Abstract

46 year old patient was admitted as an emergency with vomiting, hypotension and serum cortisol of 0,940 μg/dl (26 nmol/l) indicative of adrenal failure. Despite previous history of panhypopituitarism he was found to be hyperthyroid [free T_4 _6.32 ng/dl (ref. range: 0.93–1.7), free T_3 _22.21 pg/ml (ref. range: 1.8–4.6)]. He was fit and well till the age of 45. Eight months prior to this hospitalisation he presented with diabetes insipidus and was found to have a large cystic tumour in the area of the pituitary gland. Surgery was only partially successful and histologically the tumour was diagnosed as craniopharyngioma. Endocrine assessment revealed deficiency in ACTH-cortisol, growth hormone, and gonadotropin, as well as low-normal free T_4_. On the day of his emergency admission he looked ill and dehydrated, though was fully conscious and cooperative. Heart rate was 120 beats/min (sinus rhythm), blood pressure 85/40 mm Hg. There were no obvious features of infection, but there was marked tremor and thyroid bruit. He received treatment with intravenous fluids and hydrocortisone. L-thyroxine was stopped. Administration of large dose of methimazole (60 mg/day) resulted in gradual decrease in free T_4 _and free T_3 _(to 1.76 ng/ml, and 5.92 pg/ml, respectively) over a 15-day period. The patient was found to have increased titre of antithyroperoxidase (anti-TPO) and anti-TSH receptor (anti-TSHR) antibodies [2300 IU/l (ref. range <40) and 3.6 IU/l (ref. range <1.0), respectively]. He was referred for radioactive iodine treatment. Iodine uptake scan performed prior to radioiodine administration confirmed uniformly increased iodine uptake consistent with Graves' disease.

Our case illustrates coexistence of hypopituitarism and clinically significant autoimmune thyroid disease. The presence of hypopituitarism does not preclude the development of autoimmune thyrotoxicosis.

## Background

Adrenal insufficiency of either a primary or secondary origin may culminate in a life-threatening adrenal crisis [1]. Acute stress situations or other co-existent disease can aggravate symptomatology of glucocorticoid deficiency. Such conditions usually include acute infections or cardiovascular events. It is, however, also well recognized that thyroid hormones accelerate glucocorticoid turnover, so that thyrotoxicosis may increase glucocorticoid requirements in subjects on hydrocortisone replacement [2]. We present a case where a subject with known panhypopituitarism, including incipient secondary hypothyroidism, developed florid thyrotoxicosis that, in turn, contributed to an acute glucocorticoid deficiency.

## Case presentation

### Acute presentation

A 46 year old patient was admitted as an emergency with vomiting, hypotension and serum cortisol of 0.940 μg/dl (26 nmol/l) indicative of an adrenal failure. Despite previous history of panhypopituitarism he was found to be hyperthyroid [free T_4 _6.32 ng/dl (ref. range: 0.93–1.7), free T_3 _22.21 pg/ml (ref. range: 1.8–4.6)], see Table 1.

**Table 1:** Biochemical and hormonal results of patient at the time of diagnosis, after debulking of craniopharyngioma and on the day of emergency admission.

	**Before surgery**	**After surgery (one month later)**	**On admission with adrenal crisis (Five months later)**	**Reference range**
Sodium [mmol/l]	144	139	141	135–150

Potassium [mmol/l]	3.9	4.3	3.9	3.5–5.0

Creatinine [mg/dl]	0.8	0.8	0.9	0.6–1.4

TSH [mU/l]	0.069	0.007	0.010	0.27–4.2

Free T_4 _[ng/dl]	0.952	1.32	6.32	0.93–1.7

Free T_3 _[pg/ml ]	1.87	1.52	22.21	1.8–4.6

LH [IU/l]	0.588	<0.100	not assessed	1.7–8.6

FSH [IU/l]	0.530	0.135	not assessed	1.5–12.4

Testosterone [ng/ml]	0.355	0.023	not assessed	2.8 – 8.0

8 am Cortisol [μg/dl]	7.79*	0.168**	0.940	6.2–19.4

Prolactin [ng/ml]	18.56	16.1	not assessed	3.4–24.1

*Without hydrocortisone replacement. In order to covert μg/dl to nmol/l multiply by 27.6

**Hydrocortisone omitted in the afternoon prior to measurement.

### Past Medical History

**Figure 1:**
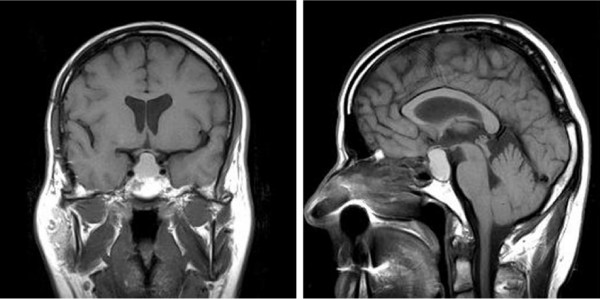
**T1-weighted magnetic resonance images of cystic tumour of the described patient.** Subsequently this was found to represent craniopharyngioma.

He was fit and well till the age of 45. His height was 183 cm, BMI 25.5 kg/m^2^. The was no family history of autoimmune disease. Eight months prior to this hospitalisation he presented with diabetes insipidus and was found to have a large cystic tumour in the area of the pituitary gland (presented in Figure 1). There was also evidence of hypopituitarism with evident hypogonadotropic hypogonadism, accompanied by relatively low 8 am cortisol, as well as low TSH and free T_4 _and free T_3 _at the lower limit of the reference range, i.e., a feature indicative of an incipient secondary hypothyroidism (Table 1). The patient was subsequently referred for transcranial surgery. Complete removal of the mass was not feasible, and so only a debulking procedure was performed. Endocrine assessment completed after his discharge from neurosurgical department revealed deficiency in ACTH-cortisol and gonadotropin axes, as well as low-normal free T_4 _(Table 1). Subsequently he was also found to have low IGF-I indicative of growth hormone deficiency. He was commenced on hydrocortisone (20 mg in the morning+10 mg at noon), oral desmopressin (0.2 mg tds), fortnightly intramuscular testosterone and L-thyroxine (25 μg od). His clinical condition remained stable for about five months, i.e., till about 4 weeks prior to the emergency hospitalisation. At that time he developed palpitations, and lost about 4 kg of weight. Over this period his condition gradually deteriorated, with increased ill-being and eventual vomiting.

On the day of his emergency admission he looked ill and dehydrated, though was fully conscious and cooperative. Heart rate was 120 beats/min (sinus rhythm), blood pressure 85/40 mm Hg. There were no obvious features of infection, but he was found to have marked tremor and thyroid bruit. Mild lid-lag sign was present, however, without any obvious orbitopathy. He was found to have very low cortisol, with markedly raised free T_4 _and free T_3_, as presented in Table 1. His full blood count revealed haemoglobin of 14.1 g/dl, white cell count of 5.27 × 10^3^/μl, with normal differential and serum osmolality 281 mOsmol/kg H_2_O. His remaining biochemical results on the day of his acute admission are also presented in Table 1.

### Treatment and Progress

**Figure 2:**
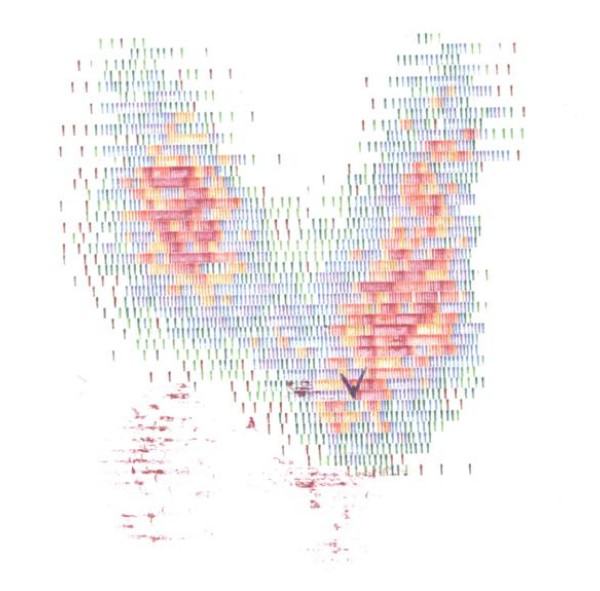
**Iodide uptake scan of the described patient prior to radioiodine administration.** Uniformly increased uptake plus raised titre of anti-TPO and anti-TSHR antibodies were indicative of Graves' disease as the cause of thyrotoxicosis.

He received treatment with intravenous fluids and hydrocortisone initially 100 mg intravenously every 8 hours for the first 24 hours. L-thyroxine was stopped. Administration of large dose of oral methimazole (60 mg/day) resulted in gradual decrease in free T_4 _and free T_3 _(to 1.76 ng/ml, and 5.92 pg/ml, respectively) over a 15-day period, as presented in Table 2. He was found to have increased titre of antithyroperoxidase (anti-TPO) and anti-TSH receptor (anti-TSHR) antibodies [2300 IU/l (ref. range <40) and 3.6 IU/l (ref. range <1.0), respectively]. Both thyroid hormones and anti-TPO and anti-TSHR antibodies were measured by standard commercial assay kits provided by Roche Diagnostics, Indianapolis, USA. He was subsequently referred for treatment with radioactive iodine. Iodine uptake scan, performed prior to radioiodine administration, confirmed uniformly increased iodine uptake consistent with Graves' disease (Figure 2). Two months after administration of radioactive iodine, he required further debulking of craniopharyngioma. At that point he was found to have low free T_4 _[0.84 ng/dl (ref. range: 0.93–1.7)], and free T_3 _[1.0 pg/ml (ref. range: 1.8–4.6)], indicative of radioiodine-induced hypothyroidism. He was recommenced on L-thyroxine, the dose of which was gradually increased to 100 μg daily. Since then his condition remains stable.

**Table 2:** Thyroid function tests on the day of an emergency admission of patient and during his hospital stay prior to transfer for radioactive iodine treatment.

**Date**	**TSH **[mU/l] (Ref. Range: 0.27–4.2)	**Free T_4 _**[ng/dl] (Ref. Range: 0.93–1.7)	**Free T_3 _**[pg/ml] (Ref. Range: 1.8–4.6)
11.02.04	0.010	6.32	22.21

16.02.04	-	3.78	10.18

20.02.04	<0.005	2.77	7.93

26.02.04	-	1.76	5.92

## Discussion

Our case illustrates development of autoimmune thyrotoxicosis in the setting of known previous panhypopitutarism. From clinical viewpoint, the most important factor is to recognise that such combination of two opposite conditions, i.e., thyrotoxicosis despite previous secondary hypothyroidism is indeed possible. Coexistence of hypopitutarism and thyrotoxicosis, though rare, has been described in literature. Some of these patients, however, developed serious complications, like thyrotoxic coma [3], or radioiodine-induced thyroid storm [4]. In the latter case Graves' disease developed eight years after the documentation of hypopituitarism in a patient also diagnosed with a craniopharyngioma. More recently a possibility of coexistence of TSH-secreting pituitary adenomas and autoimmune hypothyroidism [5], a coexistence of Graves' disease and macroprolactinoma [6] or Graves' disease and lymphocytic hypophysitis [7] have been described.

There is also evidence of significantly raised prevalence of thyroid disorders in patients with acromegaly, regardless of the preservation of other hormonal axes [8,9]. This includes a large Polish series of about 360 patients with acromegaly, where the presence of goitre was observed in 59.2% of cases, while overt hyperthyroidism developed in 8.6% of patients [8].

The patient described above had coexistent nanhypopituitarism and clinically significant autoimmune thyroid disease. Thyroid hormones are known to increase the turnover of cortisol. Our patient developed marked thyrotoxicosis during the course of Graves' disease, but failed to increase his hydrocortisone replacement. The combination of thyrotoxicosis in the setting of increased cortisol requirements had lead to a vicious circle resulting in life-threatening adrenal crisis.

## Conclusion

In summary, we have demonstrated that even severe hypopituitarism does not preclude the development of autoimmune thyrotoxicosis. These conditions are therefore not mutually exclusive and may indeed coexist in the same patient. Our case, therefore, highlights the need for awareness of possible coexistence of these conditions among endocrinologists, neurosurgeons and general physicians.

## Consent

Patient's consent have been obtained for publication of this case report.

## Competing interests

The authors declare that they have no competing interests.

## Authors' contributions

KCL, MM, E S-J and AL personally supervised treatment of the above patient at the time of diagnosis of craniopharyngioma, during his acute admission and during his follow-up in endocrine clinic. All contributed to writing of the paper. JM supervised radioiodine treatment of the patient and contributed to writing of the manuscript.
